# P01.01. Neural responses to the mechanical characteristics of a spinal manipulation: effect of varying segmental contact site

**DOI:** 10.1186/1472-6882-12-S1-P1

**Published:** 2012-06-12

**Authors:** J Pickar, W Reed, C Long, G Kawchuk

**Affiliations:** 1 Palmer Center for Chiropractic Research, Davenport, USA; 2 University of Alberta, Edmonton, Canada

## Purpose

A goal of our laboratory is to identify mechanisms of action operative during the body-based practice of spinal manipulation. Spinal manipulation can be identified by a number of mechanical characteristics, including but not limited to, contact site, magnitude, rate and direction of thrusting force. Because neural mechanisms are thought to contribute to its clinical effects, we studied spinal manipulation during a series of experiments aimed at identifying mechanical characteristics that affect responses from sensory neurons innervating paraspinal tissues. Presumably, specific parameters related to these characteristics are related to successful clinical outcomes. In this study, we determined the effect of contact site on manipulation-induced neural activity of proprioceptive afferents from lumbar paraspinal muscles.

## Methods

In an anesthetized cat preparation, a simulated spinal manipulation [posterior-to-anterior; thrust amplitude = 21.3N (55% of an average cat’s body weight of 3.95 kg); thrust duration = 100ms] was delivered to the intact lower lumbar spine (L_6_ – S_1)_ at each of 4 contact sites: L_6_ spinous process, left L_6_ mammillary process, left L_6_ lamina, and L_7_ spinous process. Electrophysiological recordings from individual muscle spindle afferents (n=16) innervating the L_6_ multifidus and longissimus muscles were obtained from L_6_ dorsal rootlets exposed through an L_5_ laminectomy. Changes in neural activity during the manipulative thrust were compared between the four contact sites.

## Results

All contact sites increased mean spindle activity: L_6_ spinous: 85 impulses per second (imp/s) (60, 100; lower, upper 95% CI); L_6_ lamina: 104 imp/s (79, 130); L_6_ mammillary 80 imp/s (55, 105); and L_7_ spinous 43 imp/s (18, 68). Lamina contact produced the largest increase, but only differences between the L_7_ spinous and each L_6_ contact site were statistically significant.

## Conclusion

The data suggest that maximizing sensory input from segmental paraspinal tissues during a spinal manipulation requires specifically contacting that segmental level. In addition, a lamina contact may most effectively create the dynamic mechanical stimulus that evokes the sensory input.

